# Influence of Sampling Conditions, Salivary Flow, and Total Protein Content in Uric Acid Measurements in Saliva

**DOI:** 10.3390/antiox8090389

**Published:** 2019-09-11

**Authors:** Jorge M. González-Hernández, Lorena Franco, David Colomer-Poveda, Silvia Martinez-Subiela, Ramón Cugat, José J. Cerón, Gonzalo Márquez, Luis M. Martínez-Aranda, Pedro Jimenez-Reyes, Asta Tvarijonaviciute

**Affiliations:** 1 Neuromove Research Group, Faculty of Sport, Catholic University of San Antonio (UCAM), Campus de los Jerónimos, 30107 Murcia, Spain; dcolomer@ucam.edu (D.C.-P.); gmarquez@ucam.edu (G.M.); lmmartinez2@ucam.edu (L.M.M.-A.); 2 Faculty of Health Science, Universidad Europea de Canarias, la Orotava, 38300 Tenerife, Spain; 3 Interdisciplinary Laboratory of Clinical Analysis, University of Murcia, 30100 Murcia, Spain; lorena.franco2@um.es (L.F.); silviams@um.es (S.M.-S.); jjceron@um.es (J.J.C.); asta@um.es (A.T.); 4 Garcia Cugat Foundation, 08023 Barcelona, Spain; montse.garcia@sportrauma.com; 5 Centre for Sport Studies, Rey Juan Carlos University, Fuenlabrada, 28943 Madrid, Spain; Peterjr49@hotmail.com

**Keywords:** squat, salivettes, biomarkers, oxidative stress

## Abstract

Uric acid (UA) is the most abundant antioxidant compound in saliva and one of the most sensitive biomarkers for detecting changes in the oxidative status of the organism. The aim of this study was to evaluate the effect of: (i) different methods of saliva sampling and (ii) the correction by salivary flow or total protein on UA concentrations in saliva. Paired saliva (collected by two different methods, passive drooling and using Salivette cotton rolls) and serum samples were obtained from 12 healthy men after the performance of two resistance training exercises of different level of effort that can produce different concentrations in UA in saliva. There were no significant differences between values of uric acid in saliva using Salivette and passive drool. Correlations between UA in serum and saliva and increases in UA in saliva after exercise were detected when saliva samples were obtained by passive drool and Salivette and were not corrected by salivary flow or total protein concentration. Therefore for UA measurements in saliva it would not be recommended to normalize the results by salivary flow or protein concentration. This study highlights the importance of choosing an adequate sampling method selection as well as the expression of results when analytes are measured in saliva.

## 1. Introduction

Saliva, as a sample where different biomarkers which can be easily measured, has attracted attention of the scientific community in recent years. The easy and painless collection are the main advantages of using saliva compared to serum use. In addition, it is a non-invasive procedure allowing its use in field conditions and repeated sampling [1], producing minimum discomfort and anxiety.

Saliva has an antioxidant system consisting of various metabolites and enzymes [2]. For this reason, saliva has been used to investigate possible changes in the oxidative status associated with different conditions, such as oral diseases [3,4], systemic diseases (e.g., diabetes [5] or renal failure [6]), and physical efforts [7]. Uric acid (UA) is the most important antioxidant molecule in saliva, contributing around 70% of the total salivary antioxidant capacity [8] and being able to chelate transition metals and to react with biological oxidants such as hydroxyl radicals, hypochlorous acid, and reactive nitrogen species [9]. In a previous report, UA was the only analyte showing a significant change when a panel of oxidative stress biomarkers was measured in saliva after an acute session of resistance exercise. Therefore, UA is one of the most important biomarkers that should be analyzed in saliva in order to evaluate oxidative stress [7].

One of the causes that can produce changes in oxidative status and increases in UA is resistance training (RT), which is a physical exercise modality allowing the modification of variables such as number of sets, repetitions, or rest time [7].Training to failure (TF) is characterized by the inability to complete a concentric phase due to the high level of fatigue [10,11]. On the other hand, training consisting of a number of repetitions that are less than the achievable maximum is known as training not leading to failure (TNLF) and has been shown to produce less fatigue and similar or even better adaptations than TF [12].

The features of the device used for sampling, as well as the way the analytes are expressed in saliva, may influence the results. For example, Salivette cotton rolls significantly increase salivary testosterone and estradiol levels [13] and in the specific case of dehydroepiandrosterone, the cotton interference effect is of sufficient magnitude to attenuate the association between serum and saliva levels [14]. Furthermore, in some analytes, such as the salivary alpha-amylase (sAA), the use of normalized values by considering salivary flow or protein concentration can produce variability in the results of sAA in different stress models, compared with the results expressed without any correction [15]. Saliva has been collected for measuring levels of UA by different methods, such as flow stimulation by chewing on paraffin [2], using a cotton roll [16], or without stimulation [17]. However, there are no comparative studies about whether different sampling and normalization procedures might affect the UA values in saliva. Therefore, the main objective of this study was to evaluate how two different sampling conditions (using passive drool or Salivette with cotton) and three different normalization procedures (without any correction, corrected by salivary flow, or corrected by total protein concentration) could influence the measured values of UA in saliva. For this purpose, the effect of different sampling conditions and normalization procedures in saliva on UA concentrations, and their correlations with serum values, were evaluated in saliva samples that were obtained by two experimental models of acute exercise (TLF and TNLF) in order to get samples with different UA concentrations.

## 2. Materials and Methods

### 2.1. Experimental Approach to the Problem

Two different squat resistance-training protocols were designed to obtain samples with different UA concentrations. Prior to the protocol performance, two familiarization sessions with saliva extraction and full squat technique training were performed, in addition to the 10 repetition maximum (10 RM) load determination [18]. First protocol included 6 sets of 10 repetitions (TF), while the second one included 6 sets of 5 repetitions (TNLF), both with 5 min of rest and the same external load corresponding to 10 repetition maximum (RM). Subjects performed two different protocols in a counterbalanced order during two consecutive weeks. Moreover, all training sessions were supervised by a strength and conditioning specialist, in order to ensure the correct performance of the exercise. On the other hand, saliva sampling was made by two different procedures, passive drool and Salivette cotton roll, both sampled for a 1 min duration. Saliva results were expressed without any correction, and corrected by salivary flow rate and total protein.

### 2.2. Subjects

Twelve trained males (age 23.5 ± 3.5 years, height 1.77 ± 0.10 m, and body mass 73 ± 7.2 kg), volunteered to participate in this study. The criteria of inclusion were the absence of any health problems or musculoskeletal injures and also the absence of ingestion of any drug in the last six months. Participants were fully informed of any possible risks and discomforts associated with experimental procedures and provided a written informed consent to participate in this study, which was approved by the local ethical committee in agreement (1349/2016) with the Declaration of Helsinki.

### 2.3. Procedures

Three training sessions were performed starting with a pre-test, followed by two post-tests days (24 and 48 h). First, baseline saliva and serum sample collections were carried out in fasting conditions. Afterwards, subjects had a breakfast (juice and a toast with tomato and olive oil). One hour later, they did a standardized warm-up protocol, followed by training sessions of 50–60 min (including warm-up) which were supervised by researchers specializing in strength and conditioning training. Every session was performed in the morning at the same schedule for each individual (±1 h), under constant environmental conditions (20 °C and 60% humidity).

Saliva and blood were sampled at four different times. The first sample corresponding with the baseline was taken at 8:30 AM. The second saliva and blood samples were collected 60 min post-training. Finally, the third and fourth extraction corresponded with 24 and 48 h post-training. The order of collection was saliva by passive flow first, followed by collecting saliva using Salivette, and finally the blood sample was collected. Participants were not allowed to eat, drink coffee or caffeinated soft drinks, or consume dairy products one hour before collecting saliva samples. Furthermore, five minutes prior to saliva collection, participants were asked to rinse their mouth with clear water to avoid contaminations.

Specifically, saliva was collected by applying two different procedures (i) passive flow for 1 min under supervision, using 5 mL standard microcentrifuge polystyrene tubes with round bottoms (12 × 75 mm) (Deltalab, 5 mL, Barcelona. Spain) [15], (ii) Salivette cotton roll (Sarstedt, Nümbrecht, Germany). Salivette rolls were chewed for 1 min and then placed into special centrifugation tubes. Each sample was refrigerated or stored on ice until arrival to the laboratory, which was no longer than 45 min after sample collection. All the samples were firstly weighed and then centrifuged at 4500× *g* for 10 min at 4 °C. The supernatant from whole saliva samples obtained by passive flow and saliva samples from the Salivettes were transferred to 1.5 mL Eppendorf tubes and stored at −80 °C until analysis. In all cases the volume of saliva obtained was 1 mL minimum.

After saliva collection, blood extractions were performed from the antecubital vein (5 mL) into one plane tube to get serum (approximately 2 mLs) that was stored at −80 °C until analysis.

UA was measured using a colorimetric commercial kit (Uric acid, Beckman Coulter Inc., Fullerton, CA, USA) following the International Federation of Clinical Chemistry (IFCC) method. This assay showed in saliva and serum less than 10% inter and intra-assay imprecision and was linear in the linearity test under dilution.

Salivary flow rate was obtained by dividing the saliva volume by the time of the sampling period (1 min) [15,19]. Saliva volume was obtained by subtracting the empty tube weight from the saliva-filled one, and values in grams obtained were considered equivalent to milliliters. UA amount was later multiplied by flow rate (mg/min).

Saliva total protein quantification (TP) expressed in mg/mL was done through a commercially available colorimetric kit for measuring urine and low-complexity region (LCR) proteins (protein in urine and CSF, Spinreact, Spain). This assay showed in saliva less than 15% inter and intra-assay imprecision and was linear in the linearity test under dilution. To express the values of UA normalized by protein content, in each sample the results from UA were divided by its total protein value (mg/mg).

### 2.4. Statistical Analyses

Data are presented as Mean (interquartile range). Data distribution was examined for normality using the D’Agostino and Pearson omnibus normality test. When data was not normally distributed, non-parametric tests were used. Uric acid values obtained in serum, and saliva using Salivette and passive droll methodologies, were compared using the Friedman test followed by Dunn’s multiple comparison test. Correlation between different variables were evaluated using the Spearman correlation test. Statistical analyses were performed using a computer software (Graph Pad Prism Version 7 for Windows, Graph Pad software, La Jolla, CA, USA). The level of significance was set at *p* < 0.05.

A post hoc power analysis was conducted using the values obtained to verify the null hypothesis. By using the mean and standard deviation of uric acid for basal time and after 60 minutes of exercise, and a power of 80% at a 5% level of significance, the number of individuals were calculated. The data analysis was done using ClinCal statistic analyzer software (https://clincalc.com/stats/samplesize.aspx).

## 3. Results

The power analysis test indicates that 10 subjects were required in order to obtain a power of 80% with a 5% level of significance.

Results of UA concentrations in serum and saliva when collected with Salivette or by passive drool, corrected either by salivary flow rate, by total protein concentration, or without any correction are shown in Table 1.

A significant increase in serum values of UA was detected at 60 min (*p* = 0.011) and 24 h (*p* = 0.009) after the TF protocol. However, no significant changes in serum uric acid were detected at any time after the TNFL protocol, although there was a tendency to increase at 24 and 48 h.

In saliva, significant increases in UA concentration without any correction were found at 60 min after the TF protocol with the two tested sampling methods (Salivette, *p* = 0.017 and passive drool, *p* = 0.021). When values were corrected by salivary flow, an increase at 60 min was also found after the TF protocol with the same two sampling methods (Salivette, *p* = 0.032 and passive drool, *p* = 0.01). After correction by total protein, a decrease in the values of UA after TF was found.

After the TNLF protocol, UA concentrations increased in saliva at 60 min (*p* = 0.011) when using passive drool, but a decrease was noticed when the values were corrected by salivary flow rate. The Salivette method revealed an increase in UA at 48 h (*p* = 0.026), and this change was not observed when the results were corrected by flow or total protein concentration. Even after correction by total protein, a decrease in the values of UA after TF was found.

In Table 2 appears the comparison between uric acid values obtained in serum and in saliva using Salivette and passive droll. There were no significant differences between the values of uric acid in saliva using Salivette and passive droll.

Correlation analysis results are depicted in Table 3. Data analysis revealed a low but significant correlation between serum and saliva UA values obtained with Salivette methods and expressed without any correction (*r* = 0.4344; *p* < 0.001) and with saliva values obtained by passive drool and expressed without any correction (*r* = 0.3728; *p* = 0.031).

## 4. Discussion

In this study we evaluated whether the sampling and normalization procedures may influence the results of UA in saliva. We used the RT model since it has been previously reported that this procedure of acute exercise increases UA in saliva [7]. The rationale for including two resistance exercise protocols of different intensities in this study is because we postulated that the different intensities could produce different values of UA in saliva. Therefore, with this design we were able to study how the procedures of saliva collection and normalization of values behave for a wide range of UA values.

We used a commercially available assay for the UA measurements in saliva since it was easy and cheap to obtain and set-up. This assay provided adequate results of imprecision and linearity under dilution when validated in saliva and serum, and did not require any modification for the salivary measurements compared to serum.

Our results showed that the TF protocol produced a significant increase in serum and saliva UA at 60 min post-training, being recovered after 48 h of exercise cessation. This is in agreement with a previous report [7], where authors reported salivary and serum UA increments 10 min after an acute bout of RT performed against 75% of one repetition maximum (10 reps × 3 sets). In contrast, we did not observe any significant increase in serum after the TNLF protocol. The increase of UA after intense exercise could be due to an increased purine oxidation and subsequent UA production [16] but it could be also protective in terms of increased antioxidant capacity of the saliva. According to our results, it can be postulated that the exercise representing maximum effort (TF) leads to a significant and fast increase of UA in serum which tends to decrease after 24 h. However, a less fatiguing protocol (such as TNLF) seems to produce lower rates of oxidative stress. Overall, the TF model allowed us to evaluate our procedures of saliva collection and normalization with different UA values.

Concerning the sampling and normalization procedures, our results revealed that values of UA in saliva obtained with Salivette and passive drool and without any correction showed a moderate correlation with serum. These results are in agreement with the ones obtained in the previous reports where Salivette was used in 54 individuals with normal weight [16] and in study with 83 subjects where passive drool was used [20]. However, the correlation coefficient (*r*) that we obtained was lower than the one reported by Kondakova et al. [18] (*r* = 0.76). These differences could be related to different conditions of the assays used or the existence of a different dynamic in the UA response to exercise in serum than in saliva. Overall, it would be desirable to evaluate a larger population to assess the correlations between passive drool and Salivette and serum. In addition, in case of using other ways of obtaining saliva such as ascorbic acid stimulation, it would be of interest to evaluate its possible effect on UA, specially taking in consideration that ascorbic acid is an antioxidant agent.

The use of Salivette has been previously recommended in some studies since, by using the rolls and after centrifugation, the saliva contains less mucins and loses viscosity, making sample processing easier [21]. However it can alter the composition of some analytes, and therefore the number of analytes that can be measured with this procedure is limited [13,22]. In our study no differences were found between the use of Salivette and passive drool in UA in saliva, so apparently both methods could be used for UA measurements.

Regarding the different normalization procedures used in this study, non-corrected UA values showed the highest correlation with serum. When the results were corrected by flow or total protein concentration, the changes on UA after exercise were generally not correlated to those obtained in serum. This is in agreement with a previous study where uncorrected values described more accurately the changes observed in saliva alpha-amylase under different experimental conditions [15]. Therefore, our results reinforce previous recommendations about not normalizing samples to protein concentration in saliva when assessing the level of physical stress or exertion. However, our findings should be also interpreted carefully since they have been obtained in specific experimental conditions and their generalization could be highly speculative. Furthermore, researchers interested in using UA measurements in saliva in other exercise protocols or clinical situations may also be well advised to reassess these comparisons as a pilot study in the laboratory using their own assay conditions and experimental procedures.

## 5. Conclusions

Overall, this report highlights the importance of an adequate sampling method selection as well as the expression of results when analytes are measured in saliva, and also stresses the need to follow standardized sampling procedures to give high-quality analytical results in these situations. It is important to point out that the use of saliva for UA measurements can have important practical applications. By monitoring the UA in saliva, situations in which there is an increase in oxidative stress can be detected and identified and appropriate measures could be taken in order to reduce or compensate the oxidative stress status. Overall, saliva can be easily used for routine evaluation of the UA level if the sample is properly collected and result adequately expressed.

## Figures and Tables

**Table 1:** Mean (interquartile range) data of serum and salivary uric acid (UA) before (Pre) and after 60 min (Post_60), 24 h (Post_24), and 48 h (Post_48) of acute exercise. TLF: training leading to failure, TNLF: training not leading to failure, TP: total proteins.

Sample Type	TLF (6 × 10)				TNLF (6 × 5)			
	Pre	Post_60	Post_24	Post_48	Pre	Post_60	Post_24	Post_48
Serum (mg/dL)	5.36 (4.08–6.61)	5.92 (4.85–6.99) *	5.97 (4.58–6.81) **	5.58 (4.34–6.18)	4.98 (4.18–6.28)	4.81 (4.22–6.14)	5.37 (4.27–6.16)	5.38 (3.87–5.92)
Salivette (mg/dL)	2.10 (1.76–3.11)	2.73 (2.25–3.53) *	2.23 (1.44–2.62)	2.17 (1.88–3.14)	1.74 (1.61–2.42)	1.97 (1.56–2.77)	1.99 (1.50–2.57)	2.29 (1.66–3.00) *
Salivettex Flow Rate (mg/min)	4.16 (3.14–5.49)	4.98 (3.52–6.84) *	3.01 (2.42–5.13)	4.09 (2.92–5.71)	3.64 (3.14–4.49)	5.10 (3.10–6.62) **	3.15 (2.51–4.31)	3.98 (3.50–4.82)
Salivetter/TP (mg/mg)	5.80 (5.08–6.83)	3.65 (2.28–4.35) *	5.05 (3.15–6.98)	4.65 (3.40–5.85)	6.64 (4.98–8.89)	2.64 (1.91–4.64) ***	4.67 (3.49–9.01)	5.47 (4.41–8.06)
Passive Drool (mg/dL)	2.47 (1.30–3.07)	2.77 (1.86–4.85) *	2.52 (1.48–2.84)	2.28 (0.96–2.51)	1.92 (1.02–2.69)	2.40 (1.27–2.95) *	1.86 (1.46–2.56)	1.73 (1.33–2.64)
Passive Drool × Flow Rate (mg/min )	2.54 (1.16–4.41)	4.51 (1.80–8.89) *	3.57 (1.58–4.14)	2.15 (0.90–3.81)	1.80 (1.24–3.02)	1.75 (1.21–4.03)	2.20 (1.37–3.78)	2.27 (1.27–3.56)
Passive Drool/ TP (mg/mg)	6.61 (3.37–8.58)	5.86 (3.19–9.15)	5.68 (2.86–10.40)	4.42 (2.31–16.6)	1.92 (1.02–2.69)	2.40 (1.27–2.95) *	1.86 (1.46–2.56)	1.73 (1.33–2.64)

* *p* < 0.05 **p < 0.01, *** *p* < 0.001 vs. Pre of the same group.

**Table 2:** Comparison between uric acid values obtained in serum and in saliva using Salivette and passive drool.

Analyte	Serum	Salivette	Passive Drool
Uric Acid, mg/dL	5.38 (4.25–6.36)	2.11 (1.67–2.76) *	2.17 (1.40–2.84) *

* *p* < 0.001 vs. levels in serum.

**Table 3:** Correlation data between uric acid in serum and saliva.

Sample type	Spearman *r*	95% Confidence Interval	*p* Value
Salivette	0.4344	0.2506–0.5880	<0.001
Salivette Flow	0.1795	−0.02787–0.3720	0.080
Salivette/TP	0.1676	−0.04009–0.3614	0.102
Passive Drool	0.3728	0.07919–0.4605	0.031
Passive Drool Flow	0.3541	0.1131–0.4871	0.089
Passive Drool/TP	0.09	−0.3365–0.4859	0.170
